# A Case Report on Adenosquamous Carcinoma of Gallbladder: A Very Rare Malignancy

**DOI:** 10.7759/cureus.15791

**Published:** 2021-06-21

**Authors:** Shobha Mandal, Sravan K Ponnekanti, Chandrakala Dadeboyina, Anusha Tipparthi, Vineela Kasireddy

**Affiliations:** 1 Internal Medicine, Guthrie Robert Packer Hospital, Sayre, USA; 2 Internal Medicine, Osmania Medical College, Hyderabad, IND; 3 Hematology and Oncology, Guthrie Robert Packer Hospital, Sayre, USA

**Keywords:** gallbladder neoplasm, adenosquamous carcinoma, dna heterogeneity, squamous, adenosquamous, gallbladder, carcinoma

## Abstract

Gallbladder (GB) carcinoma is a rare carcinoma with a poor prognosis. The prevalence is 0.7-21/100,000 worldwide and 1-2/100,000 in the United States. Adenosquamous cell carcinoma is composed of glandular and squamous components. The overall five-year survival rate is less than 5%, with a median survival of fewer than six months. We are presenting a case of adenosquamous carcinoma of the GB in a 76-year-old female who presented with right upper quadrant abdominal pain and was found to have an enlarged GB, with thickened irregular wall likely inflammatory or malignant and mildly dilated common bile duct on ultrasound imaging of the abdomen. Core needle biopsy of GB showed findings compatible with adenosquamous carcinoma and immunohistochemistry was positive for P40, CK5,6. She was diagnosed with stage T4 N0 M0. She was started on chemotherapy with cisplatin and gemcitabine (25 mg/m2 and 1000 mg/m2), respectively, every three weeks but her condition worsened after the fifth cycle of chemotherapy and she decided to move forward with hospice care given her bad prognosis. Unfortunately, she passed away one week after being discharged home.

## Introduction

Gallbladder (GB) carcinoma is of rare occurrence among GI malignancies. Trend analysis from the Surveillance, Epidemiology, and End Results (SEER) database indicates an increase in the incidence of late-stage GB carcinoma in the United States. In 2018, about 219,000 people were estimated to have been diagnosed with GB cancer [1-2]. Because of the lack of specific signs and/or symptoms for GB cancer, deep anatomical location of the organ, late clinical presentation, and diagnosis it gets diagnosed in the late stage and has an overall poor prognosis [2]. Here we present a patient who was diagnosed with adenosquamous GB carcinoma, a rare GI malignancy with a poor prognosis.

## Case presentation

A 76-year-old female with a history of endometrial cancer treated with total abdominal hysterectomy and bilateral salpingo-oophorectomy presented to the primary care physician with the complaint of right upper quadrant abdominal pain, which was colicky in nature, gradual in onset, progressively getting worse over the last six months, well-controlled with acetaminophen once daily. She also complained of pale-colored stools, dark yellow urine, and yellowish discoloration of her skin over the last six months. She denied any change in her weight, changes in her bowel habit (constipation or diarrhea), blood in the stool, heartburn, nausea, or vomiting. She never smoked, drank alcohol, or used illicit drugs. Her mother died of complications of breast cancer at the age of 38. 

**Figure 1:**
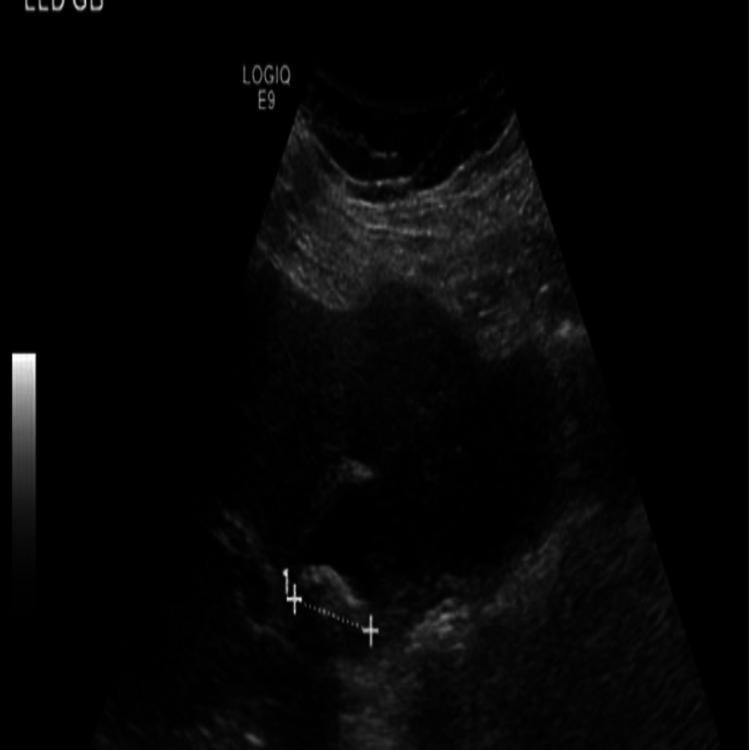
Ultrasound of the abdomen showing enlarged gallbladder, with thickened irregular wall likely inflammatory or malignant.

**Figure 2:**
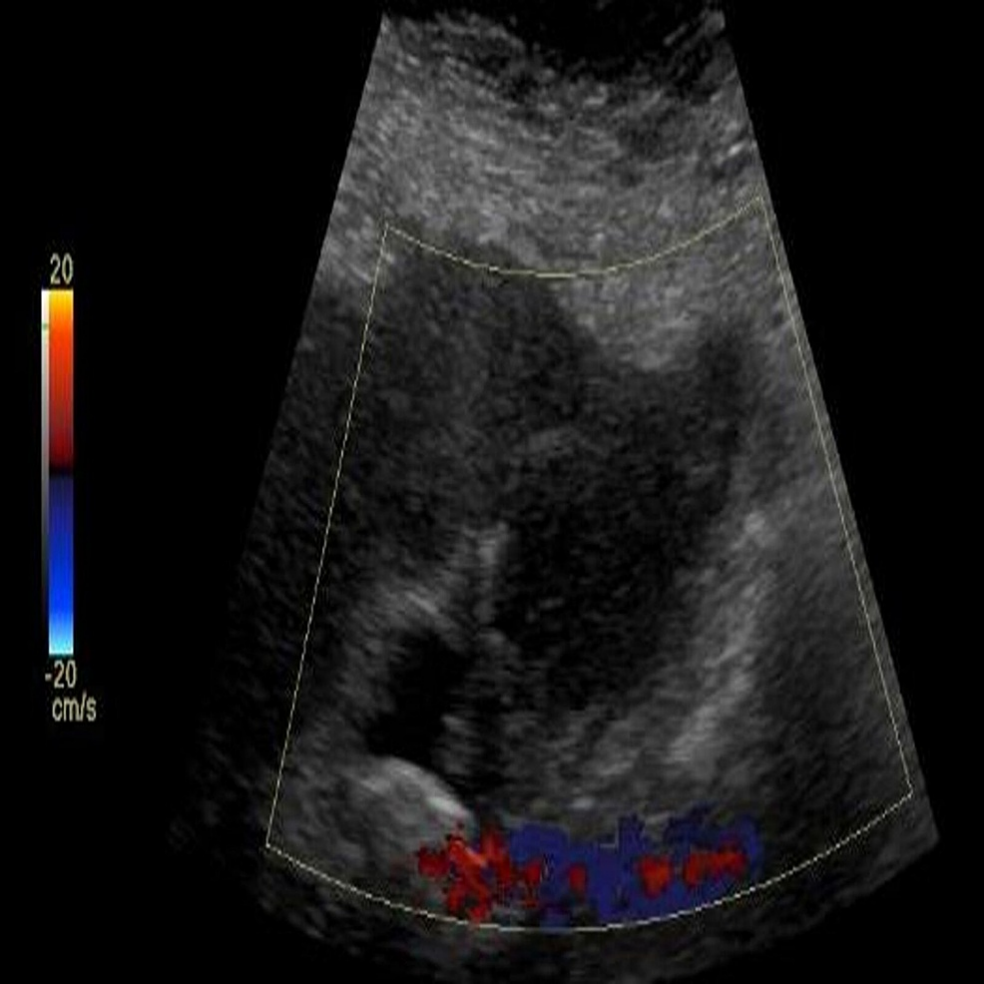
Ultrasound of the gallbladder showing enlarged gallbladder, with thickened irregular wall.

On physical examination, she was vitally stable, bilateral scleral icterus was present, and all other exams including abdomen were normal. Laboratory studies showed mild leukocytosis (17000 per microliter) with neutrophilia (83%). Liver function test results were within normal limits but bilirubin was elevated (2.9 mg/dL). Ultrasound of the abdomen revealed enlarged GB, with thickened irregular wall likely inflammatory or malignant and mildly dilated common bile duct (Figures 1 and 2).

**Figure 3:**
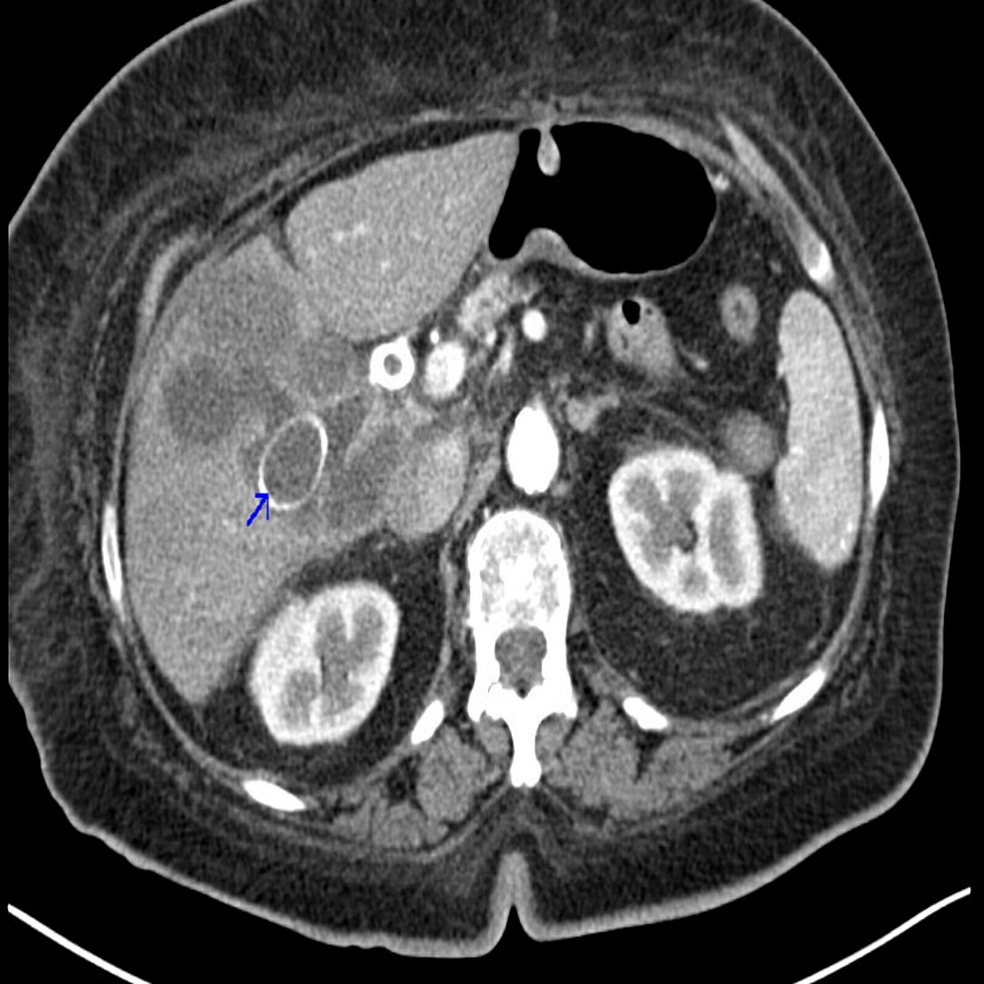
CT scan of the abdomen showing a large necrotic appearing mass.

Contrast-enhanced CT scan of the abdomen revealed a large necrotic appearing mass measuring 5.4 x 5.9 x 6.1 mm causing mass-effect on the GB, extending into the liver and the second portion of the duodenum (Figure 3).

**Figure 4:**
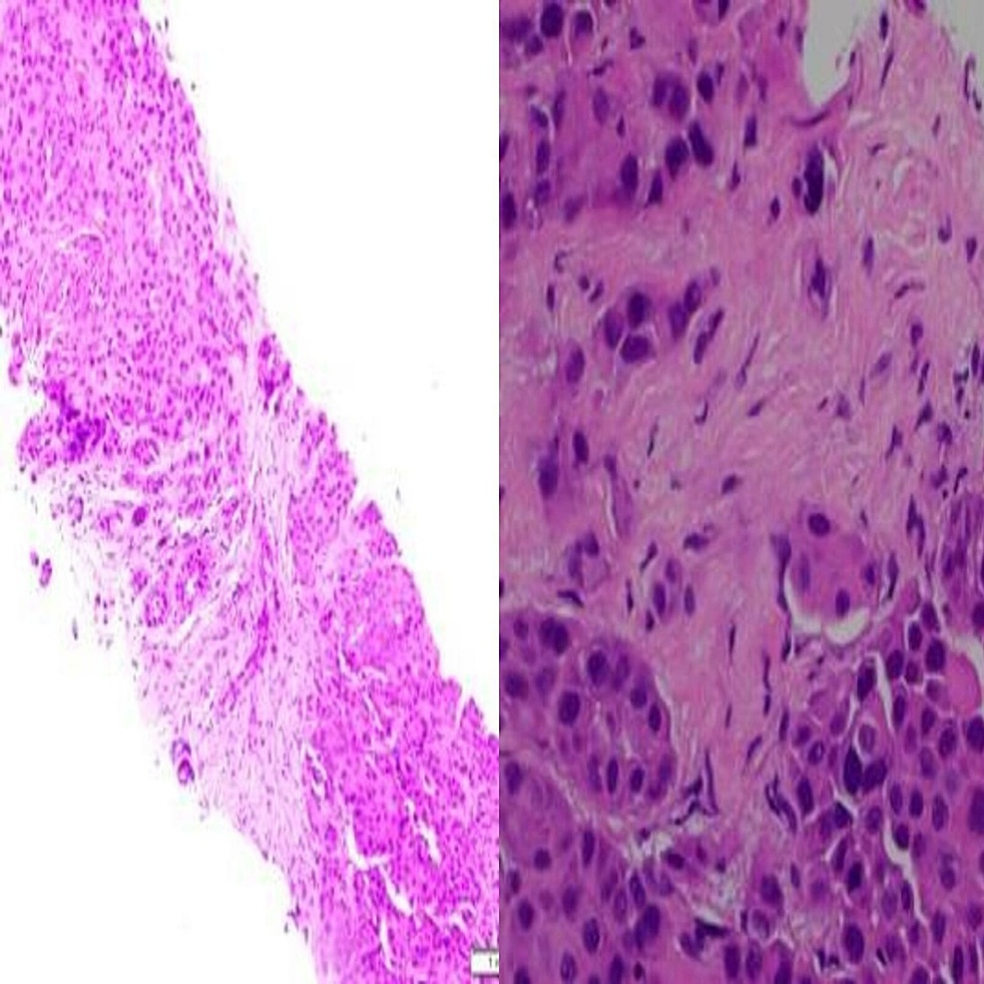
Core needle biopsy showing adenosquamous carcinoma of the gallbladder (magnification: left image 10X, right image 40X).

As the abdominal imaging was suspicious for malignancy vs inflammatory process, core needle biopsy of the mass was performed which showed findings suggestive of adenosquamous carcinoma (Figure 4).

**Figure 5:**
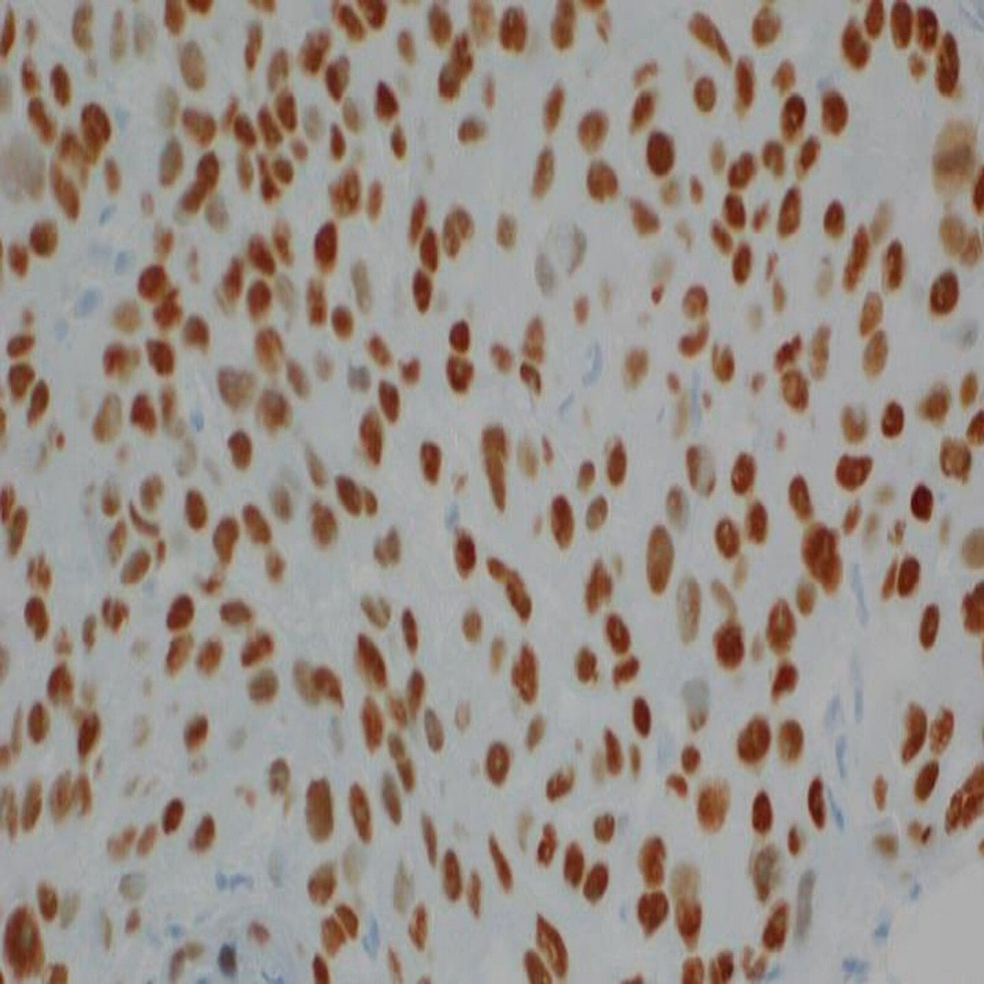
Immunohistochemistry of tumor cells positive for P40.

**Figure 6:**
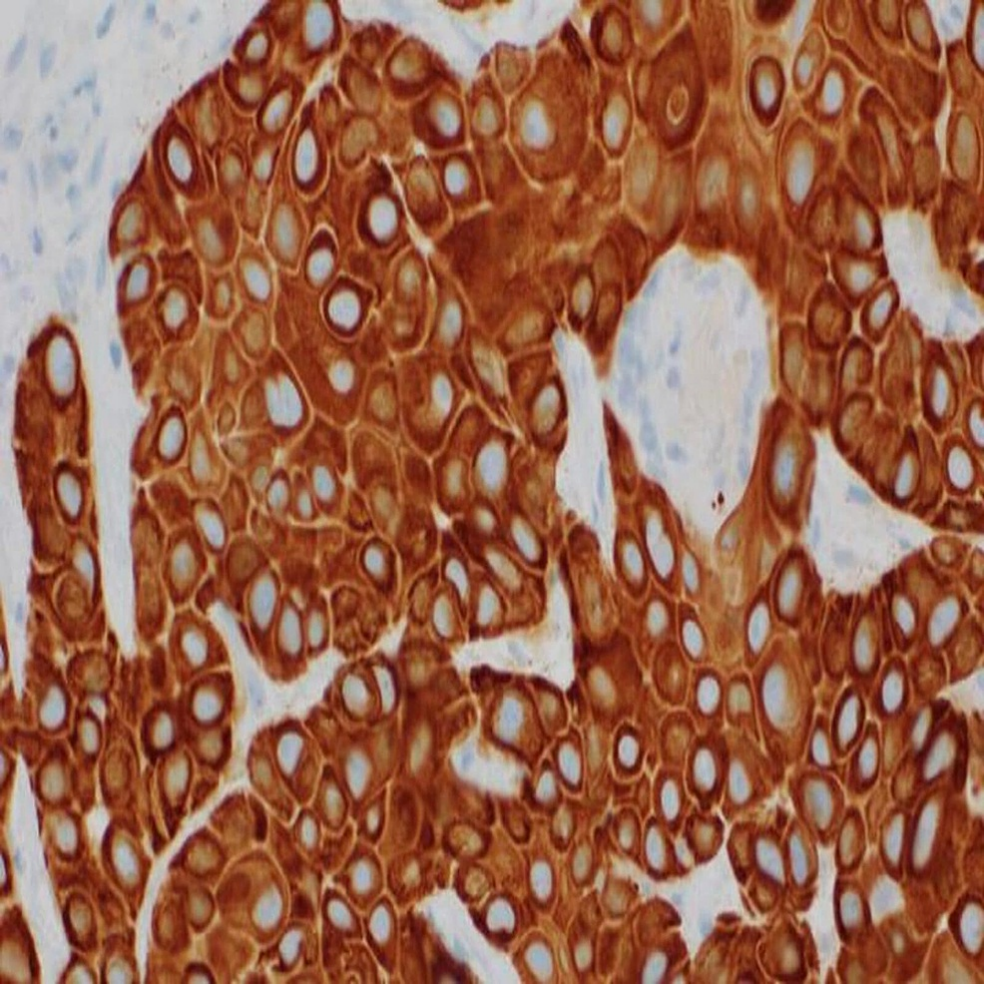
Immunohistochemistry of tumor cells positive for CK5/6.

**Figure 7:**
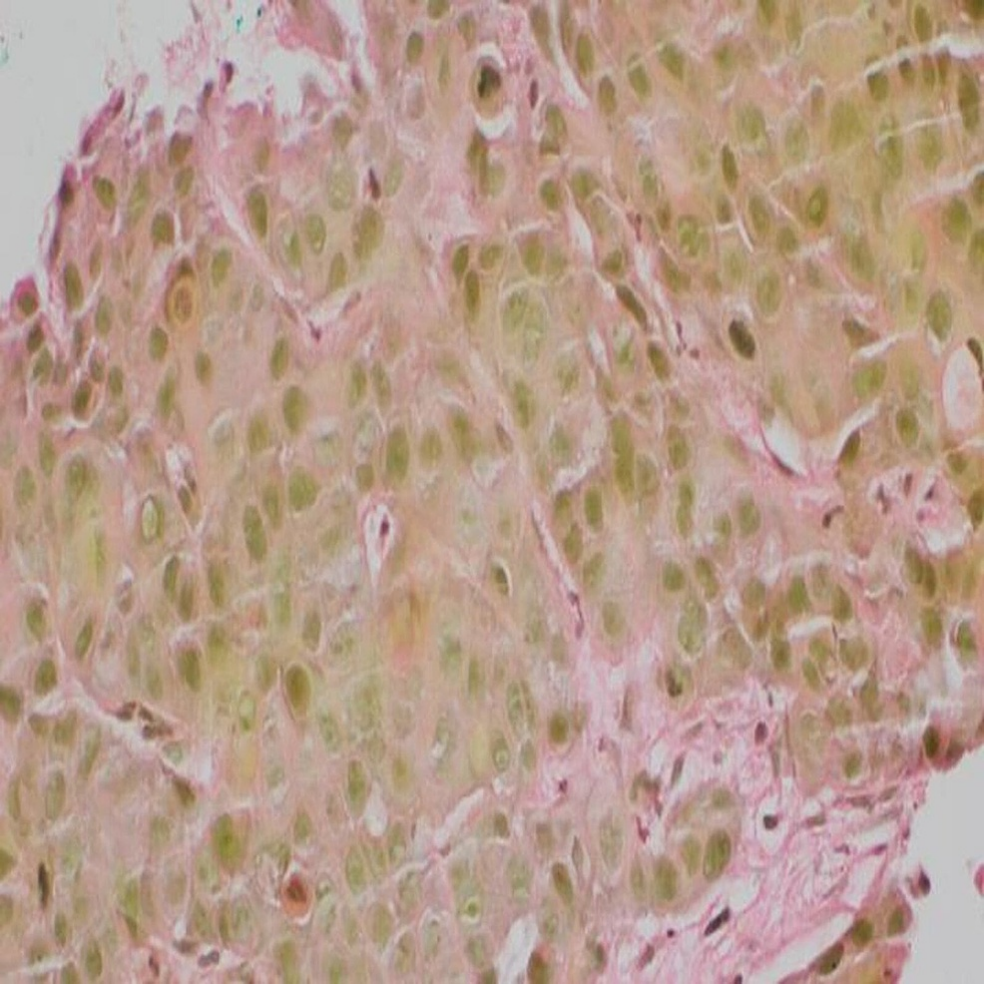
Immunohistochemistry of tumor cells negative for mucicarmine stain.

Immunohistochemistry of the tumor cells was positive for P40 (Figure 5) and CK5/6 (Figure 6) and was negative for mucicarmine stain (Figure 7).

CA 19-9 tumor marker was also elevated (2839 U/mL). For further staging and to rule out other sources of foci of the tumor, the patient underwent positron emission tomography/CT (PET/CT) which showed fluorodeoxyglucose (FDG) avid large necrotic mass adjacent to the GB in the region of porta hepatis causing some obstruction and extending up to hepatic flexure and, duodenum, with central biliary ductal dilatation. She underwent endoscopic retrograde cholangiopancreatography (ERCP) which showed an extrinsic stricture of the common hepatic duct 1.5 cm distal to the bifurcation and underwent biliary sphincterotomy and metal stenting. The tumor was staged cT4 N0 M0.

She was evaluated by a medical and surgical oncologist. A decision was made to start her on neoadjuvant therapy first and reevaluated again in a few months for resection as the size of the mass was large and was involving contiguous structures. She was started on chemotherapy with cisplatin 25 mg/m2 and gemcitabine 1000 mg/m2 every three weeks. Restage scan after completion of three cycles of chemotherapy showed interval enlargement of large necrotic mass in GB fossa measuring 7.5 x 7.6 x 8.0 cm but tumor markers were trending down and she reported improvement in her symptoms hence chemotherapy was continued.

After completion of the fifth cycle, she had worsening fatigue and new onset of nausea and vomiting. She was also found to have septic shock secondary to urinary tract infection. Laboratory workup showed leukocytosis, elevated creatinine 1.9 mg/dl (0.7-1.2 mg/dl ) and lactic acid 3.2 mmol/L (0.7-2.1 mmol/L). She was admitted to acute intensive care and was started on an empiric antibiotic with parenteral broad-spectrum antibiotic vancomycin and piperacillin-tazobactam. Repeat CT chest, abdomen and pelvis showed small to moderate right pleural effusion, large GB stone with poor visualization of the GB, possible fluid and air collections measuring 5.2 x 3.4 cm, a perihepatic fluid collection with air locules, concerning for abscess or infection. She underwent emergent biliary stenting and drainage of the large perihepatic abscess. Despite appropriate medical treatment, she continued to worsen with multiorgan failure. Palliative care was on board and there was extensive discussion regarding the goals of care with the patient and family. The patient decided to go ahead with hospice care.

## Discussion

GB carcinoma is a rare, aggressive cancer with a poor prognosis. It affects females more than males and the ratio is 3:1. The prevalence of GB carcinoma is 0.7-21/100,000 worldwide and 1-2/100,000 in the United States [1]. GB carcinoma affects all ethnicities and geography but the incidence is the highest in Northern India, Pakistan, East Asia, Eastern Europe, and South America including Columbia and Chile [2]. Factors that increase the risk of GB carcinoma are chronic inflammation, cholelithiasis, porcelain GB, GB polyp, primary sclerosing cholangitis, chronic infection with salmonella, Helicobacter pylori, congenital biliary cysts, and abnormal pancreaticobiliary duct junction. Rarely, medications such as isoniazid, oral contraceptive pills, and methyldopa also increase the risk. It most commonly affects postmenopausal females, who are cigarette smokers and obese [3]. Different histological subtypes of primary GB carcinoma in the descending order are adenocarcinoma (90%), adenosquamous (5-10%), squamous cell carcinoma (1-6%), and oat cell carcinoma [4]. Adenosquamous cell carcinoma is composed of glandular and squamous components. The squamous component is between 25 and 99%. Any tumors with less than 25% squamous component are known as adenocarcinoma with focal squamous change and those with no glandular component are known as pure squamous cell carcinomas [5].

Adenosquamous carcinoma is a moderately differentiated neoplasm composed of glandular (mucin) and squamous (keratin) components [6]. In multiple studies, it is demonstrated that the squamous component of adenosquamous GB carcinoma has a greater proliferative capacity and it grows twice as fast as glandular components [7]. Squamous cell carcinoma of the GB spreads to local organs because of its potential to directly invade the adjacent organs and local lymph nodes. Adenocarcinoma has more metastatic spread potential compared to squamous [8]. GB carcinoma most commonly invades the liver but can also involve the duodenum, stomach, colon, pancreas, and extrahepatic bile duct [9-12]. GB carcinoma patients usually remain asymptomatic until advanced stage pT3 or pT4 [13] and are found incidentally on imaging of the abdomen when worked up for other reasons. Any patient who becomes symptomatic presents with a complaint of right upper quadrant abdominal pain, anorexia, nausea, vomiting, and palpable liver mass [14].

Ultrasound is the initial choice of imaging in the evaluation of GB pathologies. Patients with GB mass or pathologies suggestive of malignancy should undergo staging workup for the local and distant spread. CT abdomen and pelvis together with MRI with cross sectionals and arterial phase images can be considered for staging and determine the resectability of the tumor. GB carcinoma appears as a mass replacing the GB in 40-65%, focal or diffuse GB wall thickening in 20-30%, and as an intraluminal polypoid mass in 15-25% of cases [15]. MRI is used to better understand the involvement of the hepatoduodenal ligament, portal vein encasement, and lymph node [15]. The tumor, node, metastasis (TNM) staging system of the combined American Joint Committee on Cancer (AJCC)/Union for International Cancer Control (UICC) is now the preferred classification scheme. Also, the staging of the tumor depends on the proliferative index of the patient, if they have a higher proliferation index in squamous components of adenosquamous carcinomas it may account for a higher T stage [16]. 

Treatment modalities depend on the stage of cancer, performance status of the patient, and side effects of treatment but in the case of GB carcinoma we have limited therapeutic options because of the aggressive nature of the carcinoma. Curative surgical resection with cholecystectomy can be considered in the patient with minimal invasion of the local organs who have a tumor limited to the GB wall without the involvement of the lymph nodes or adjacent structures in the hepato-duodenal ligament [17]. Early-stage tumors with liver invasion can be treated with liver resection together with cholecystectomy followed by systemic or regional chemotherapy. Patients treated with radical resection of the tumor were found to have better outcomes compared to the resection of the primary tumor [18]. Adenosquamous and squamous cell carcinoma of the GB appear more suitable for resection than does adenocarcinoma. Those patients who are in advanced stages where curative resection is not possible are found to have minimal benefits despite treatment with surgical resection and systemic chemotherapy. Recently newer modalities like targeted therapy and chemoradiation are also tried for the treatment of patients with GB carcinoma [17,18]. The prognosis of all the patients is guarded even after resection of adenosquamous or squamous cell carcinoma of the GB because of direct extension and multiple organ invasion. Prognosis depends upon factors such as histologic type, histologic grade, and stage of the tumor. The overall five-year survival rate of adenosquamous carcinoma is less than 5% with a median survival of fewer than six months [19].

## Conclusions

In conclusion, GB carcinoma is extremely rare and has a poor prognosis because of limited treatment options. Further research and clinical trials are warranted for better management of patients with adenosquamous carcinoma of the GB. Reporting this rare variant adds to the literature and helps in better understanding this rare tumor.
